# Supplementary material for: Cluster magnetic octupole induced out-of-plane spin polarization in antiperovskite antiferromagnet
Source: Nat Commun. 2021 Nov 11;12:6524. doi: 10.1038/s41467-021-26893-6 (PMC8585975; doi:10.1038/s41467-021-26893-6)
Supplement: Supplementary file 1 — Supplementary Information [file 41467_2021_26893_MOESM1_ESM.pdf]

Supplementary information

Figure S1 shows the surface morphology of the 34 nm Mn_3SnN (110) film, indicating that the whole film is continuous and smooth. The average surface roughness *Ra* is 0.199 nm.

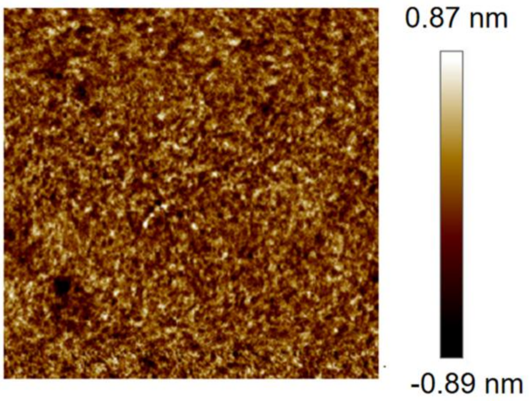

Fig. S1 Surface morphology of the film with an area of 2 μm × 2 μm.

Figure S2 shows the magnetic property of the 34 nm Mn_3SnN film after subtracting the diamagnetic background of the MgO substrate at 300 K. The film shows a small magnetization of ~16000 A/m, which is also discovered in other noncollinear AFM films[S1-S5].

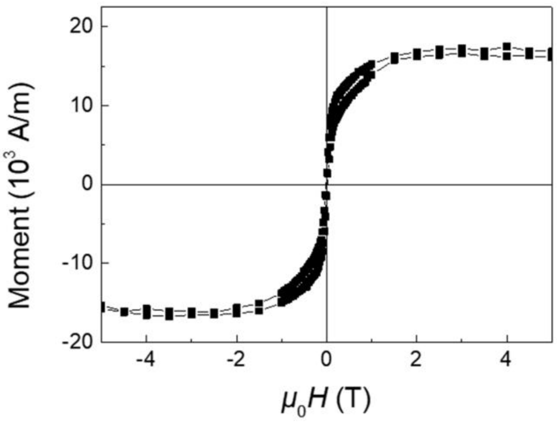

Fig. S2 Magnetization hysteresis loops of 34 nm Mn_3SnN (110) film deposited on MgO (110) substrate without diamagnetic background from the substrate. Out-of-plane magnetic field was applied at 300 K.

Figure S3 shows the ST-FMR measurement of (001)-oriented Mn_3SnN(16 nm)/Py(12 nm) sample at room temperature, where the magnetic mirror plane is represented by the dashed black line. When the current *I* is applied along the [100] or [010] directions (shown in Figs. S3a and S3d, respectively), which has parallel component to the cluster magnetic octupole moment, we can observe apparent ST-FMR signals from $\boldsymbol{\sigma}_\mathbf{z}$ in Figs. S3b, S3c, S3e and S3f. For the case in Fig. S3a, the antidamping and field-like spin torque ratios of $\boldsymbol{\sigma}_\mathbf{z}$, $\theta_{\mathrm{AD},z}$ and $\theta_{\mathrm{FL},z}$ are 0.017±0.001 and 0.090±0.003, respectively. For the case in Fig. S3d, the antidamping and field-like spin torque ratios of $\boldsymbol{\sigma}_\mathbf{z}$, $\theta_{\mathrm{AD},z}$ and $\theta_{\mathrm{FL},z}$ are 0.019±0.002 and 0.123±0.006, respectively, comparable to the values of the case in Fig. S3a. Note that the values of the extracted torque induced by $\boldsymbol{\sigma}_\mathbf{z}$ is also close to that of (001)-orientated Mn_3GaN, where $\theta_{\mathrm{AD},z}$ and $\theta_{\mathrm{FL},z}$ are 0.019 and 0.15, respectively[S6].

Differently, for the case in Fig. S3g, where the current is applied perpendicular to the cluster magnetic octupole moment, the antidamping and field-like spin torque ratios of $\boldsymbol{\sigma}_\mathbf{z}$, $\theta_{\mathrm{AD},z}$ and $\theta_{\mathrm{FL},z}$ are 0.001±0.001 and 0.016±0.004, respectively, much smaller than that of Figs. S3a and S3d. A quite weak $\boldsymbol{\sigma}_\mathbf{z}$ still exists, which may be caused by the slight misalignment of the device or/and the imperfect growth of the film. In a word, the ST-FMR results of (001)-oriented Mn_3SnN also support our conclusions in the main text.

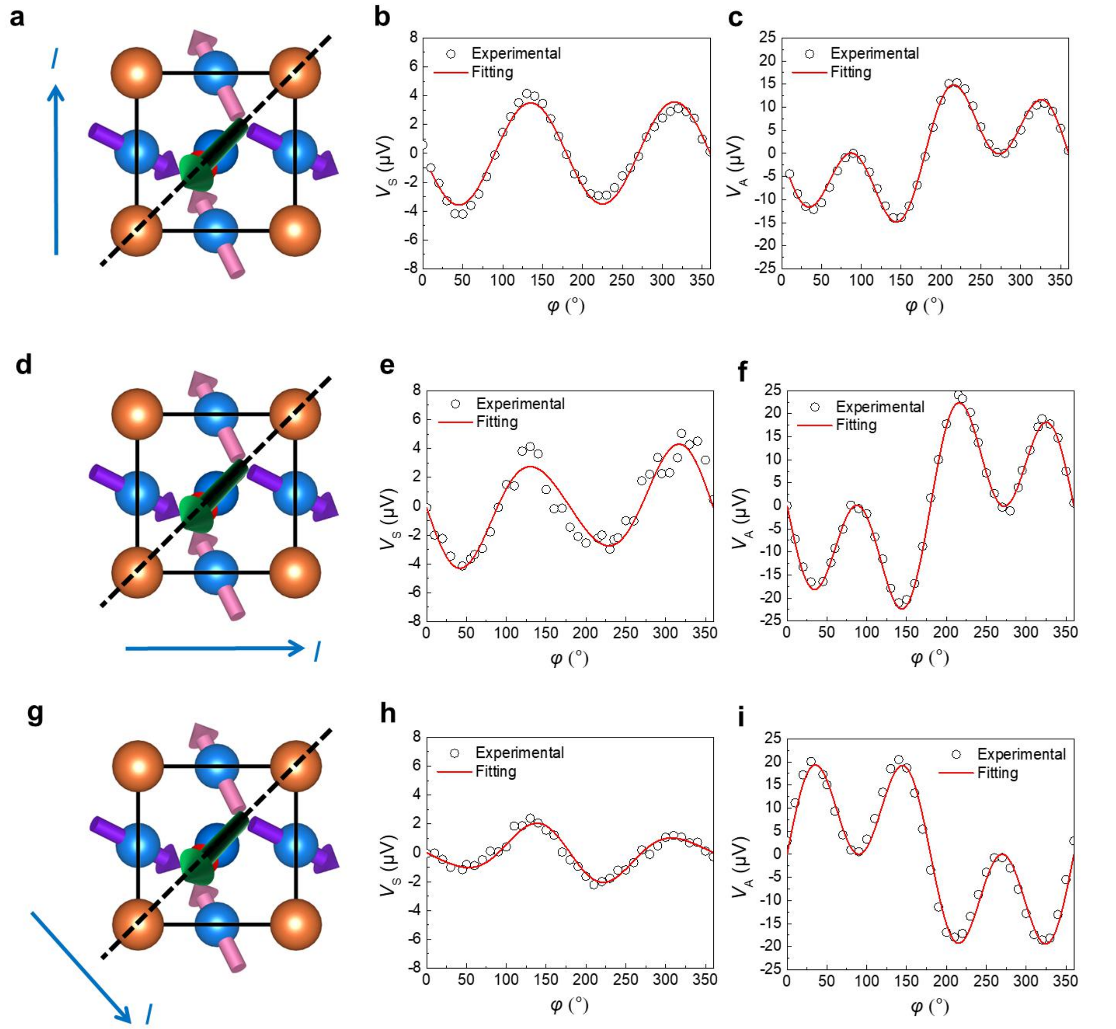

Fig. S3 ST-FMR measurement of (001)-oriented Mn_3SnN/Py sample at room temperature. **a** Schematic diagram of magnetic structure and current direction, where the magnetic mirror plane is represented by the dashed black line. The current is applied along the [100]-direction. Angular dependence of line shape amplitude of ST-FMR signals for **b** symmetric and **c** antisymmetric signal in Mn_3SnN/Py structure. **d** Schematic diagram for the case when the current is applied along the [010] direction. Angular dependence of line shape amplitude of ST-FMR signals for **e** symmetric and **f** antisymmetric signal in the same sample. **g** Schematic diagram for the case when the current direction is perpendicular to the cluster magnetic octupole moment. Angular dependence of line shape amplitude of ST-FMR signals for **h** symmetric and **i** antisymmetric signal in the same sample.

Figure S4 displays the ST-FMR measurement of the (110)-oriented Mn_3SnN/Py sample at 380 K (the highest of our equipment). We can see that $\boldsymbol{\sigma}_\mathbf{z}$ still exists since 380 K is lower than the Néel temperature (475 K) of Mn_3SnN. The antidamping and field-like spin torque ratios of $\boldsymbol{\sigma}_\mathbf{z}$, $\theta_{\mathrm{AD},z}$ and $\theta_{\mathrm{FL},z}$ at 380 K are 0.003±0.001 and 0.018±0.003, respectively, smaller than the values at room temperature. The existence of $\boldsymbol{\sigma}_\mathbf{z}$ but with smaller spin torque ratios supports $\boldsymbol{\sigma}_\mathbf{z}$ is related to the magnetic configuration (the cluster magnetic octupole).

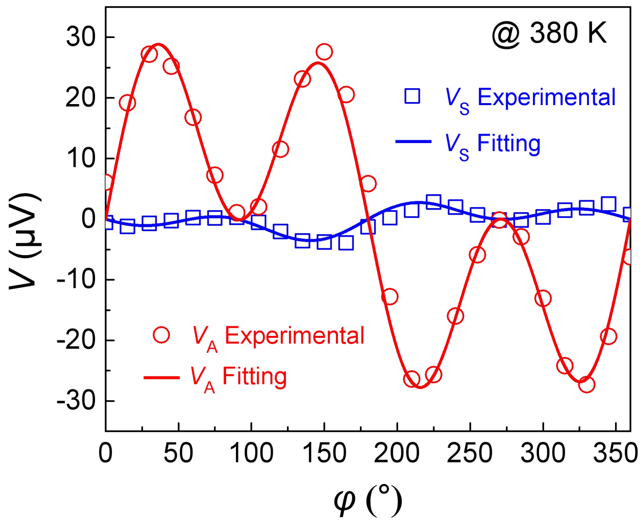

Fig. S4 ST-FMR measurement of the (110)-oriented Mn_3SnN/Py sample at 380 K. The blue or red curve represents the angular dependence of line shape amplitude of ST-FMR signals for symmetric or antisymmetric signal, respectively.

Figure S5 shows the measurement configuration of the resistivity of Mn_3SnN and $Mn_3SnN/(Co/Pd)_3$ samples at room temperature. The resistivity of Mn_3SnN film deposited on the MgO substrate is 913.1±0.2 μΩ cm and the resistivity of $(Co/Pd)_3$

multilayer is 74.8±0.1 μΩ cm based on the simple parallel resistance formula. Then the current density to achieve the field-free SOT switching is estimated to be ~9×10^6 A cm^{-2}, taking the film thickness of Mn_3SnN(12 nm)/(Co(0.4 nm)/Pd(0.8 nm))$_3$ sample, the channel width of 5 μm and the average critical switching current of ~28 mA into account.

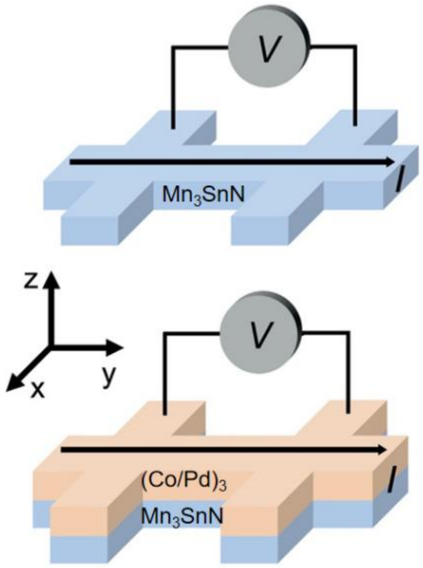

Fig. S5 Measurement configuration of the resistivity of Mn_3SnN and $Mn_3SnN/(Co/Pd)_3$ at room temperature.

Figure S6 illustrates typical field-free SOT switching for 10 times. Remarkably, the SOT switching does not decline after 10 cycles, revealing the robustness of our device.

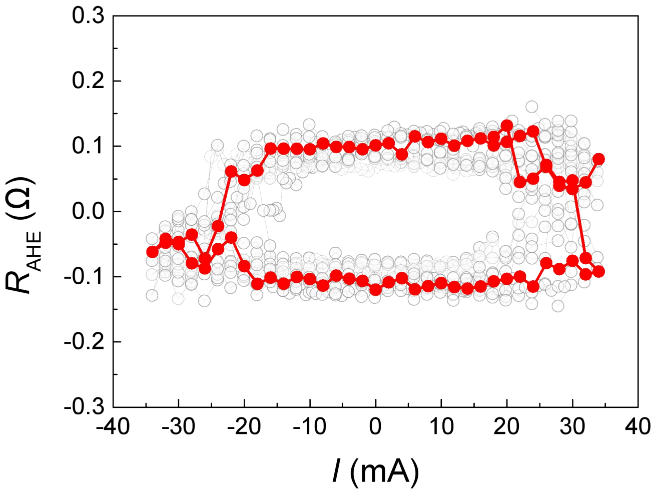

Fig. S6 Field-free SOT switching of 10 cycles. The red one is the same loop in Fig. 4c.

Figure S7 shows the in-plane *M-H* curve of Mn_3SnN(12 nm)/(Co(0.4 nm)/Pd(0.8 nm))$_3$ sample we use to realize the field-free SOT switching. We can see that there is no exchange bias at room temperature, revealing that the field-free switching is irrelevant to the exchange bias.

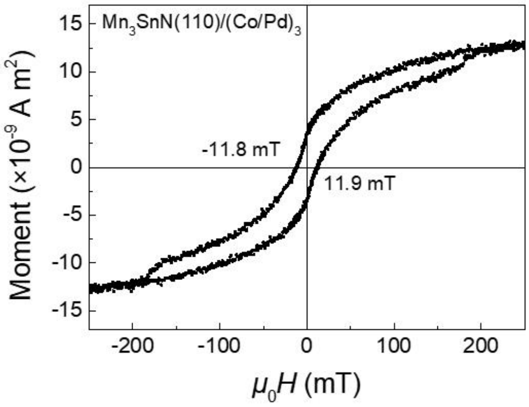

Fig. S7 In-plane hysteresis loop of Mn_3SnN(12 nm)/(Co(0.4 nm)/Pd(0.8 nm))$_3$ at room room temperature.

Figure S8 exhibits the R_{AHE} relationship with different pulse current *I* along the [001] direction under a large magnetic field of 500 mT along the same direction as *I*

(Fig. S8a), which is larger than the anisotropy field of the Co/Pd multilayer (Fig. S8b). We observe that there is no switching signal under 500 mT, showing that the variation of anomalous Hall resistance is not triggered by the thermal effects caused by the pulse current.

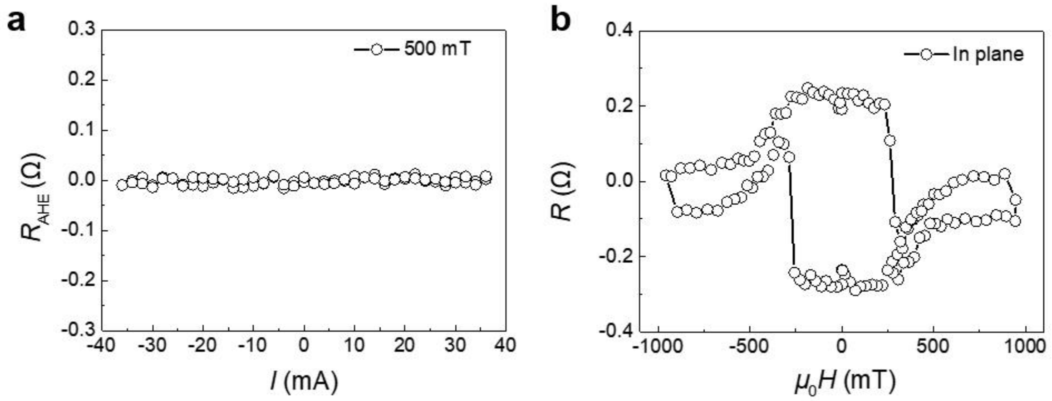

Fig. S8 **a** R_{AHE} relationship with different pulse current I when a large magnetic field of 500 mT is applied along the same direction (the [001] direction) as I. **b** R-H curve recorded with an in-plane magnetic field.

Table S1 makes a comparison of the antidamping and field-like spin torque ratios of $\boldsymbol{\sigma}_\mathbf{z}$, $\theta_{\mathrm{AD},z}$ and $\theta_{\mathrm{FL},z}$, for the present Mn_3SnN and three typical materials with $\boldsymbol{\sigma}_\mathbf{z}$, e.g., Mn_3GaN[S6], WTe_2[S7] and $MnPd_3$[S8]. Although $\theta_{\mathrm{AD},z}$ of Mn_3SnN is smaller than that of other three materials, $\theta_{\mathrm{FL},z}$ of Mn_3SnN is larger than that of WTe_2 and $MnPd_3$, reflecting that in Mn_3SnN/Py system, the out-of-plane spin polarization $\boldsymbol{\sigma}_\mathbf{z}$ mainly contributes to the field-like torque. The comparatively large field-like torque is most likely due to the spin accumulation at the Mn_3SnN/Py interface, which interacts with the adjacent Py layer, producing an exchange field[S9]. The relatively large $\theta_{\mathrm{FL},z}$ affirms the existence of $\boldsymbol{\sigma}_\mathbf{z}$ in our material, which is promising for high-density and low-power spintronics.

Table S1 Comparison of the antidamping and field-like spin torque ratios of $\boldsymbol{\sigma}_{\mathbf{z}}$, $\theta_{\mathrm{AD},z}$ and $\theta_{\mathrm{FL},z}$, for Mn_3SnN, Mn_3GaN, WTe_2 and $MnPd_3$.

Materials	$\theta_{\mathrm{AD},z}$	$\theta_{\mathrm{FL},z}$	Reference
Mn_3SnN	0.003	0.053	This work
Mn_3GaN	0.019	0.15	S6
WTe_2	0.013	0.0325	S7
$MnPd_3$	0.014	0.046	S8

Table S2 makes a comparison of the magnetization of different noncollinear AFM films. We can see that the magnitude of our Mn_3SnN film is reasonable. The weak uncompensated magnetization may be caused by the imperfect growth or the spin canting of the films. Massive researches have shown that the uncompensated magnetization is not the reason for the large anomalous Hall effect, anomalous Nernst effect or the magneto-optical Kerr effect in noncollinear AFM. In fact, the Berry curvature in momentum space of the special AFM spin texture, that is, the cluster magnetic octupole, gives rise to the above physical phenomena. The direction related $\boldsymbol{\sigma}_{\mathbf{z}}$ and field-free SOT switching here also illustrates that the weak magnetization of the film does not have obvious influence on the switching measurement.

Table S2 Comparison of the magnetization of different noncollinear AFM films.

Materials	Néel Temperature	Measurement Temperature	Magnetization	Reference
Mn_3SnN	475 K	300 K	16 emu/cm^3	This work
Mn_3Sn	420 K	300 K	34 emu/cm^3	S1
Mn_3Ge	390 K	Room temperature	34 emu/cm^3	S2
Mn_3Ga	650 K	Room temperature	48 emu/cm^3	S3
Mn_3NiN	260 K	150 K	36 emu/cm^3	S4
Mn_3GaN	345 K	300 K	7.2 emu/cm^3	S5

References

[S1] Markou, A., Taylor, J. M., Kalache, A., Werner, P., Parkin, S. S. P. & Felser, C. Noncollinear antiferromagnetic Mn_3Sn films. *Phys. Rev. Mater.* **2**, 051001 (2018).

[S2] Ogasawara, T., Kim, J., Ando, Y. & Hirohata, A. Structural and antiferromagnetic characterization of noncollinear D019 Mn_3Ge polycrystalline film. *J. Magn. Magn. Mater.* 473, 7-11 (2019).

[S3] Kurta, H., Rode, K., Tokuc, H., Stamenov, P., Venkatesan, M. & Coey, J. M. D. Exchange-biased magnetic tunnel junctions with antiferromagnetic ε-Mn_3Ga. *Appl.*

Phys. Lett. 101, 232402 (2012).

[S4] Boldrin, D. et al. The anomalous Hall effect in non-collinear antiferromagnetic Mn_3NiN thin films. *Phys. Rev. Matter.* **3**, 094409 (2019).

[S5] Hajiri, T., Ishino, S., Matsuura, K. & Asano, H. Electrical current switching of the noncollinear antiferromagnet Mn_3GaN. *Appl. Phys. Lett.* **115**, 052403 (2019).

[S6] Nan, T. et al. Controlling spin current polarization through non-collinear antiferromagnetism. *Nat. Commun*. **11**. 4671 (2020).

[S7] MacNeill, D., Stiehl, G. M., Guimaraes, M. H. D., Buhrman, R. A., Park, J. & Ralph, D. C. Control of spin-orbit torques through crystal symmetry in WTe_2/ferromagnet bilayers. *Nat. Phys.* **13**, 300–305 (2017).

[S8] DC, M. et al. Observation of anti-damping spin-orbit torques generated by in-plane and out-of-plane spin polarizations in $MnPd_3$ *arXiv preprint* arXiv:2012.09315 (2020).

[S9] Chen, X. et al. Observation of the antiferromagnetic spin Hall effect. *Nat. Mater.* **20**, 800–804 (2021).
